# Role of Intraspecies Recombination in the Spread of Pathogenicity Islands within the *Escherichia coli* Species

**DOI:** 10.1371/journal.ppat.1000257

**Published:** 2009-01-09

**Authors:** Sören Schubert, Pierre Darlu, Olivier Clermont, Andreas Wieser, Giuseppe Magistro, Christiane Hoffmann, Kirsten Weinert, Olivier Tenaillon, Ivan Matic, Erick Denamur

**Affiliations:** 1 Max von Pettenkofer-Institut für Hygiene und Medizinische Mikrobiologie, Munich, Germany; 2 INSERM U535, Hôpital Paul Brousse, Villejuif, France; 3 INSERM U722 and Université Paris 7, Paris, France; 4 INSERM U571 and Université Paris 5, Paris, France; Tufts University School of Medicine, United States of America

## Abstract

Horizontal gene transfer is a key step in the evolution of bacterial pathogens. Besides phages and plasmids, pathogenicity islands (PAIs) are subjected to horizontal transfer. The transfer mechanisms of PAIs within a certain bacterial species or between different species are still not well understood. This study is focused on the High-Pathogenicity Island (HPI), which is a PAI widely spread among extraintestinal pathogenic *Escherichia coli* and serves as a model for horizontal transfer of PAIs in general. We applied a phylogenetic approach using multilocus sequence typing on HPI-positive and -negative natural *E. coli* isolates representative of the species diversity to infer the mechanism of horizontal HPI transfer within the *E. coli* species. In each strain, the partial nucleotide sequences of 6 HPI–encoded genes and 6 housekeeping genes of the genomic backbone, as well as DNA fragments immediately upstream and downstream of the HPI were compared. This revealed that the HPI is not solely vertically transmitted, but that recombination of large DNA fragments beyond the HPI plays a major role in the spread of the HPI within *E. coli* species. In support of the results of the phylogenetic analyses, we experimentally demonstrated that HPI can be transferred between different *E. coli* strains by F-plasmid mediated mobilization. Sequencing of the chromosomal DNA regions immediately upstream and downstream of the HPI in the recipient strain indicated that the HPI was transferred and integrated together with HPI–flanking DNA regions of the donor strain. The results of this study demonstrate for the first time that conjugative transfer and homologous DNA recombination play a major role in horizontal transfer of a pathogenicity island within the species *E. coli*.

## Author Summary

The species *Escherichia coli* comprises non-pathogenic, commensal bacterial strains belonging to the normal gut microbiota of humans and many animals, but also pathogenic strains, which cause different types of intestinal or extraintestinal infections in man and animals. Single factors and mechanisms involved in pathogenesis of extraintestinal pathogenic *E. coli* (ExPEC) have been analyzed in detail for many years. The genetic information of these virulence factors has largely been acquired by horizontal DNA transfer. Key elements of horizontal transfer are large DNA fragments, called genomic islands, integrated into the conserved *E. coli* chromosomal backbone. The transfer of genomic islands within the *E. coli* species, however, has yet been elusive. In this study, we focused on the High-Pathogenicity Island (HPI), which is a genomic island widely spread among *E. coli.* It serves as a model for horizontal transfer within the *E. coli* species. We used a combination of sequenced based methods (Multi Locus Sequence Typing) and DNA–transfer experiments to decipher the transfer mechanisms of DNA–islands within the *E. coli* species. The results of this study demonstrate for the first time that conjugative transfer and homologous DNA recombination play a major role in horizontal transfer and spread of a pathogenicity island within *E. coli*.

## Introduction

The diversity of bacteria is caused by various genetic mechanisms including point mutations, genetic rearrangements and horizontal gene transfer (HGT), all of which represent driving forces of bacterial evolution [1]. While point mutations and genetic rearrangements only lead to slow evolutionary development primarily without creation of novel genetic determinants, the HGT produces extremely dynamic genomes, in which substantial amounts of DNA are introduced into and deleted from the chromosome. Thus, HGT can effectively change the life style of bacterial species. This is particularly true for bacterial pathogens, where virulence is linked to acquisition of virulence determinants by horizontal transfer and the evolution of bacterial virulence may be viewed as a process of adaptation that enables a pathogenic lifestyle. The introduction of small DNA fragments by transformation has been identified as a effective tool for HGT in natural competent bacteria [2]. However, more widespread means of HGT include the transfer of large DNA elements such as plasmids, phages and genomic islands (e.g. pathogenicity islands, PAIs) [3],[4]. These PAIs are especially important in processes leading to new bacterial pathotypes as the incorporation of a PAI can, in a single step, transform a normally benign organism into a pathogen [5]. However, the mechanisms underlying the mobilization and transfer of PAIs are still not well understood, nor is the evolution of pathogenicity islands within a certain bacterial species.

We used the “High Pathogenicity Island” (HPI) as a model to determine the evolution of a common pathogenicity island. The HPI was first detected in the plague agent *Yersinia pestis* and other highly virulent *Yersinia* species [6],[7] and encodes for a siderophore (yersiniabactin) mediated iron-uptake system, which is required for full virulence expression in *Yersinia*. Interestingly, an orthologous and highly conserved HPI is widely distributed among different species and genera of the family *Enterobacteriaceae*. The HPI reveals all structural features of a typical pathogenicity island, e.g. it is (i) integrated at a tRNA gene (*asn* tRNA), (ii) carries a gene for a phage-type integrase and (iii) displays a G-C content distinct from that of the *E. coli* chromosomal backbone. Previous studies have shown that the HPI is strongly associated with extraintestinal pathogenic *E. coli*, which cause human infections such as septicemia, meningitis, urinary tract infections and peritonitis [8],[9].

The purpose of this study was to determine the distribution of the HPI in view of the *E. coli* genomic backbone and to decipher the mechanisms of HPI transfer within the *E. coli* species. For this, we have analyzed the molecular variation of 6 representative genes of the HPI as well as of DNA loci of the *E. coli* genomic backbone directly adjacent to the HPI in *E. coli* strains from the *E. coli* reference (ECOR) collection. We then compared the phylogeny of these loci to that of the genomic backbone of each strain, which is represented by 6 housekeeping genes. The data of this nucleotide sequence based approach provided evidence for different HPI-transfer events, which include not only the HPI itself but also flanking regions of the genomic backbone. We further experimentally showed that the HPI can be transferred by conjugative F plasmid and integrate in the recipient genome by homologous recombination of flanking DNA regions. This F plasmid-mediated HPI-transfer resulted in a nucleotide sequence pattern, which provides a perfect correlate of the scenario found in ECOR strains using multilocus sequence typing (MLST) approach for both HPI genes as well as the upstream and downstream genomic sequences and chromosomal housekeeping genes.

## Materials and Methods

### Bacterial Strains

Strains of the ECOR collection were used as this set of strains is representative of the genetic diversity of the *E. coli* species [10]. The affiliation of each of the ECOR strains to the corresponding phylogenetic group has been confirmed by PCR as described [11]. Among them, all the HPI positive (37 strains) as well as 13 HPI negative strains selected for their genetic diversity and belonging to the A, B1, E and D phylogenetic groups were studied in more details (all the B2 phylogenetic group strains are HPI positive).

### Detection of HPI Genes

Detection of HPI encoded genes (*int*, *ybtQ, ybtA*, *irp2*, *irp1* and *fyuA*; Figure S1) in the strains of this study was performed by PCR amplifications with primers listed in Table S1. In addition to the PCR amplifications of HPI encoded genes, the presence of these HPI genes was verified by Southern hybridization according to standard protocols using DNA probes derived from the respective PCR products.

### Multilocus Sequencing

Different sets of primers were used for PCR amplifications and subsequent sequencing (Tables S1 and S2): (i) First, the phylogenetic relationships among the strains were inferred using MLST data from 6 housekeeping genes (*trpA, trpB, pabB, putP, icd* and *polB*), which are thought to experience little recombination and to produce a strong phylogenetic signal [12]. The sequences from these 6 genes were concatenated (5919 bp) and subjected to phylogenetic analyses. The phylogeny from these concatenates is considered, so far, as the best available *E. coli* strain (or species) phylogeny [12],[13]. (ii) Secondly, to obtain representative MLST data of the HPI from different *E. coli* isolates, oligonucleotide primers were designed based on sequences of the HPI encoded genes *int*, *ybtQ, ybtA*, *irp2*, *irp1* and *fyuA* (Figure S1). The obtained sequences were then concatenated (4119 bp). (iii) Third, the chromosomal DNA regions immediately flanking the HPI [upstream (837 bp) and downstream (1162 bp) HPI] were PCR amplified and sequenced using primers derived from the *E. coli* K-12 genomic backbone (*yeeI* gene) as well as from the genome sequence of the HPI-positive extraintestinal pathogenic *E. coli* isolate CFT073 [14],[15] (Figure S2). Sequences were trimmed to uniform length for each gene after multiple alignments and edited by using programs EditSeq, MegAlign, and SeqMan II (DNASTAR, Inc., Madison, WI, USA).

### Phylogenetic Analyses

Sequences were aligned using the Clustal program [16]. The phylogenetic analyses were performed following two steps. Trees were first obtained by the maximum likelihood (ML) procedure (PHYML software [17]) with 6 parameters of nucleotide substitution (GTR model), rate of heterogeneity of substitution among sites (with 8 discrete classes), and proportion of invariant sites. For each data set, the best-fitting model of nucleotide substitution and rate heterogeneity parameters was chosen among the 56 models proposed by MODELTEST [18] applying the maximum likelihood method through the Akaike criterion (Table S3). The bootstrap majority rule consensus trees (100 replicates) were then obtained using PHYML with the estimation of parameters under the best-fitting model previously obtained.

### Tree Comparisons

The pairwise comparisons between the resulting trees were performed as follows. Each tree *T_i_* (obtained by ML procedure) is at first expressed in term of a distance matrix *M_i_*, the pairwise distance *d^i^_ab_* between two sequences *a* and *b* being equal to the number of branches connecting them along the tree, regardless of their lengths. In doing so, this tree distance matrix allows recovering unambiguously the initial tree structure. Two tree distance matrices, e.g. *M_i_* and *M_j_* (e.g. from two different gene trees) can then be compared by estimating the path length difference metric (*pld_ij_*), i.e. the square root of the sum of squares of the differences between the elements of these matrices [19]. A weak *pld* means that the similarity between the two tree distance matrices is high and, consequently, that the tree structures are similar. Permutations can be performed to test the null hypothesis of no similarity between the tree structures [20]–[22]. As several gene tree structures are compared through the path length difference metric, a distance matrix *D* can be built, with elements being the pairwise *pld_ij_*. This matrix *D* was transformed into a tree by applying a NJ procedure [23]. To obtain a statistical confidence on this “tree of gene trees”, indicating the overall similarity between gene trees, a bootstrapping procedure was carried out as follows. First, one obtained 500 bootstrapped trees of each gene (by maximum likelihood, with PHYML), allowing to calculate 500 bootstrapped *D* matrices. Then, from these 500 *D* matrices one built 500 “trees of gene trees” by NJ procedure leading to the outcome of a consensus tree with bootstrap percentages. A high value of bootstrap proportion that delimits two set of gene trees is indicating that they are largely incongruent trees, while a low value means that they can be viewed as not incongruent.

### HPI Transfer

HPI transfer experiments were conducted as follows. First the HPI of *E. coli* strain NU14 [24] was tagged by insertion of a chloramphenicol resistance cassette into the HPI downstream the *fyuA* gene applying the method of Datsenko and Wanner [25]. The resulting strain NU14cm was mated with the F-episome carrying *E. coli* laboratory strain XL1-Blue MRF' (Te^r^) (Stratagene) resulting in a F-episome positive *E. coli* NU14cmF' strain, which was used as donor strain in the following mating experiments. Transfer of the HPI from *E. coli* NU14cmF' to recipient *E. coli* K-12 strains AB1157 and AB1157 *recA^−^* [26] was performed by biparental mating on agar plates with selection for transconjugants using chromosomal antibiotic markers of the recipient (nalidixic acid and streptomycin) and chloramphenicol. The conjugation was performed over night with a 1∶1 ratio of donor to recipient cells. Sequencing of the flanking chromosomal regions upstream and downstream the HPI in donor, recipient and transconjugant strains was performed using primers given in Table S1.

### Nucleotide Sequences

The nucleotide sequences have been submitted to GenBank and assigned the accession numbers FJ211866-FJ212161 and FJ263552-FJ263614.

## Results

### The HPI Is Widely Distributed Among Different *E. coli* Phylogenetic Groups and Inserted Mainly at the *asnT* tRNA Locus

**Figure 1:**
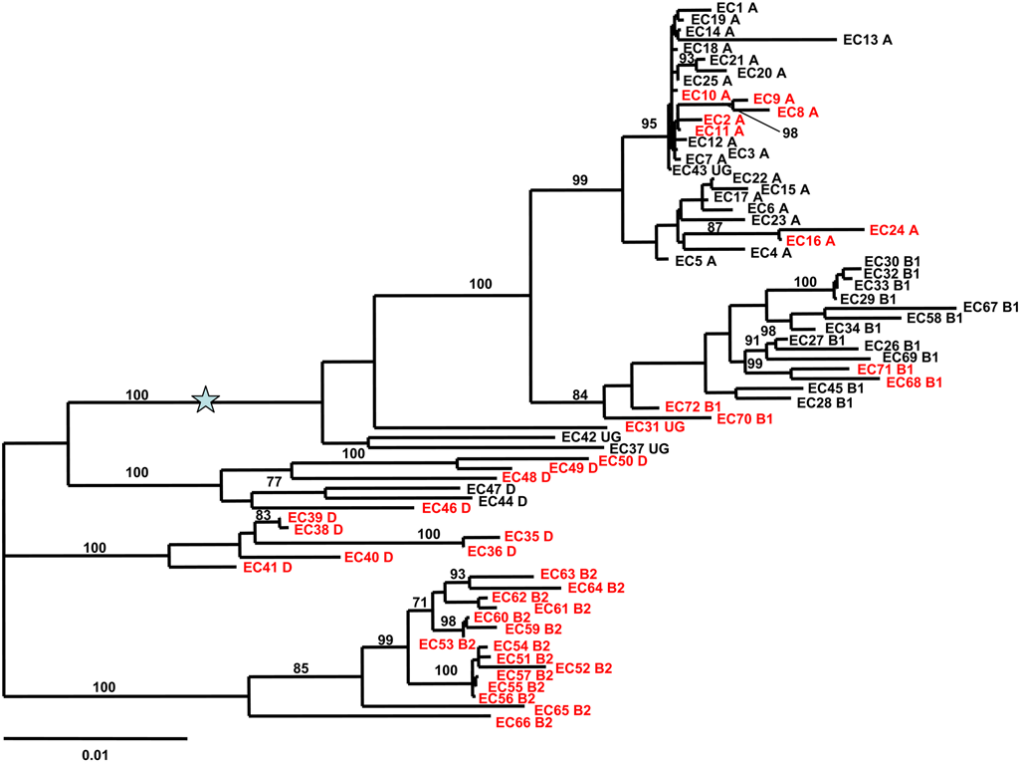
Phylogenetic unrooted tree using maximum likelihood procedure of the 72 *E. coli* strains of the ECOR collection [10]. The B2 strains are arbitrarily grouped apart and the star indicates the midpoint rooting. The tree is based on the simultaneous analysis of six chromosomal housekeeping genes (*trpA*, *trpB*, *pabB*, *putP*, *icd,* and *polB*) and represents the strain evolutionary history [12],[13]. Bootstrap values, calculated on 1,000 replicated trees, are shown if higher than 70%. The ECOR strains are indicated by EC following by their number and the phylogenetic group to which they belong (A, B1, D, B2, and UG for ungrouped) [47]. Strains given in red are HPI positive.

By means of Southern hybridization and PCR analyses for HPI encoded genes (*intB*, *ybtQ, ybtA*, *irp2*, *irp1*, and *fyuA*) we first determined the distribution and composition of the HPI among the strains of the ECOR collection. In agreement with previous results [27] we found that all strains of the phylogenetic group B2 and almost all of group D carried the HPI, whereas strains of groups A and B1 were found to be only occasionally HPI positive (Figure 1). This is consistent with the distribution of the HPI to extraintestinal pathogenic *E. coli* (ExPEC), as phylogenetic groups B2 and D predominantly consist of ExPEC strains causing urinary tract infections, septicaemia and meningitis. Interestingly, the HPI-positive strains of A and B1 carry other PAIs common to the *E. coli* strains of phylogenetic groups B2 and D [28],[29].

In all except one HPI-positive ECOR strain (ECOR31), the HPI was found to be inserted at the *asnT* tRNA gene and to carry a unique deletion of the right (*fyuA*-sited) border of the HPI leading to a loss of the direct repeat. This deletion also affects the *E. coli* genome adjacent to the HPI leading to a distinct mutation pattern in all these ECOR strains, which differs from those sequences of HPI-negative *E. coli* strains (Figure S2). We could demonstrate previously, that the ECOR31 strain carries a unique HPI of a distinct structure (ICE-type) and is located at the *asnV* tRNA copy [30].

### The HPI Is Acquired by Horizontal Transfer

**Figure 2:**
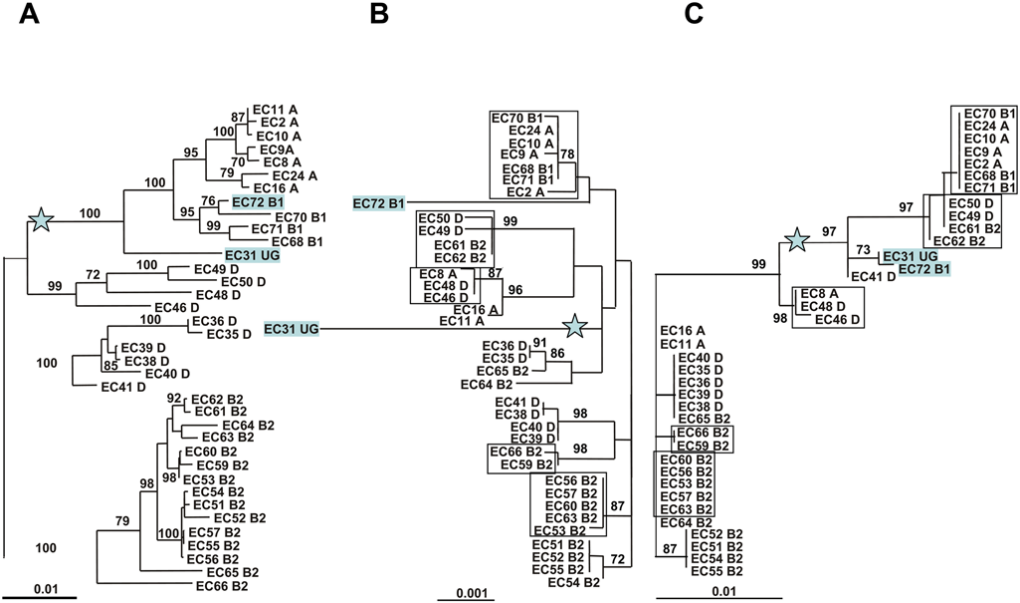
Phylogenetic unrooted trees using maximum likelihood procedure of the 37 HPI positive ECOR strains used in this study. The B2 strains are arbitrarily grouped apart and the star indicates the midpoint rooting. (A) The *E. coli* phylogenetic tree is based on the simultaneous analysis of six chromosomal housekeeping genes (*trpA*, *trpB*, *pabB*, *putP*, *icd,* and *polB*) and represents the strains' evolutionary history [12],[13]. (B) The HPI–phylogenetic tree is based on the simultaneous analysis of six genes of the HPI (*int, ybtQ, ybtA, irp2, irp1,* and *fyuA*). Strains that do group together in the HPI-phylogenetic tree, but not in the *E. coli* phylogeny tree (A) are boxed. These strains reveal identical grouping in the trees of the upstream region of the HPI (C) and the downstream region of the HPI (Figure S4). (C) The tree is based on the region upstream the HPI (UR). Strains EC31 UG and EC72 B1 are indicated in grey boxes, as these strains carry a distinct type of HPI [27],[30] (Figure S3). Bootstrap values, calculated on 1,000 replicated trees, are shown if higher than 70%. The ECOR strains are indicated by EC following by their number and the phylogenetic group to which they belong (A, B1, D, B2, and UG for ungrouped) [47].

**Figure 3:**
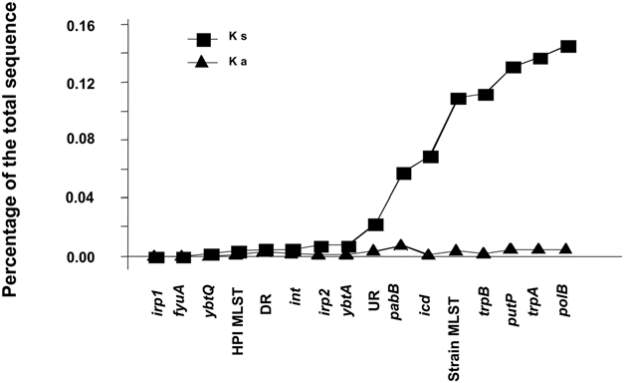
Percentage of non-synonymous (Ka) and synonymous (Ks) mutations of the studied genes in the 30 HPI positive *E. coli* strains, for which a DR region is complete. The genes are ranked according to an increase in the Ks.

The distinct distribution of the HPI among the ECOR strains represented with their phylogenetic relationships based on the MLST data of 6 housekeeping genes (Figure 1) poses the question of (i) whether this distribution is the result of multiple independent insertions of the HPI, or (ii) if a single common ancestor led to a clonal distribution, which has been affected by multiple complete HPI deletion events in single branches of the *E. coli* phylogenetic tree. To address this issue, we compared in the 37 HPI-positive ECOR strains the phylogenetic history of the *E. coli* genomic backbone represented by MLST data based on 6 housekeeping genes (Figure 2A) to the phylogenetic history of the HPI represented by the MLST data based on 6 HPI borne genes (Figure 2B). Visual inspection clearly identifies multiple incongruences between the trees. Strains belonging to different phylogenetic groups exhibit highly related HPI (Figure 2B). Furthermore, within the strains of phylogenetic group B2, the HPI from strains ECOR66 and 59 on one hand, and from strains ECOR 53, 56, 57, 60, and 63 on the other, are grouped together with high bootstrap values (Figure 2B). This is in complete disagreement with the phylogeny of these strains (Figure 2A). These data indicate that the HPI evolutionary history is clearly distinct from the strain phylogenetic history, which may indicate multiple arrivals of the HPI within the *E. coli* strains, as reported for other virulence genes [31]. Of note, long branches indicating specific evolutionary histories are observed for the HPI of strains ECOR72 and 31 (Figure 2B). As cited above, the ECOR31 strain carries a copy of the HPI, which differs from all other ECOR strains. The HPI of ECOR31 carries an additional 30 kb DNA region downstream the *fyuA* gene encoding a functional conjugative mating pair formation and DNA processing system [30]. The HPI of ECOR72 is inserted at the *asnT* tRNA, but its upstream border is deleted by insertion of an *IS*Sfl14 insertion element [27] (Figure S3). We also compared the level of polymorphism in the HPI genes and in the chromosomal backbone genes. In both categories of genes, non-synonymous mutations are rare indicating a negative selection (Figure 3). However, synonymous mutations that can be considered as a neutral molecular clock, are infrequent in the HPI as compared to the housekeeping genes (Figure 3) (Mann-Whitney, p = 0.0008). This is another strong argument for a different evolutionary history of the HPI and the backbone chromosome, with a recent arrival of the HPI in the *E. coli* species.

### The HPI Is Spread within *E. coli* Species Together with Its Flanking Backbone Chromosomal Regions

Next, we wanted to obtain some clues about the molecular mechanisms involved in the distribution of the HPI among the different phylogenetic groups of the ECOR strains. Schematically, two scenarios can be envisaged: The HPI may have been repeatedly horizontally transferred from another species to *E. coli* followed by an integration of the HPI at the *asnT* tRNA gene. Alternatively, after acquisition from a non *E. coli* species and integration at the *asnT* tRNA gene the HPI may have subsequently been spread by horizontal transfer within the *E. coli* species. A way to distinguish between these two scenarios is to characterize and compare the sequences flanking the HPI. In the first scenario, the insertion is site-specific and located next to the *asnT* tRNA. It concerns only the HPI, while the flanking regions should have the same phylogenetic history as the rest of the chromosomal background. In the alternative scenario, spreading within species is not limited to the HPI, but affects a larger region including the genomic DNA regions flanking the HPI. In this case, the phylogenetic history of the flanking regions should be the same as of the HPI. We thus sequenced roughly 1 kb of each DNA region immediate upstream (UR) and downstream (DR) the HPI (Tables S1 and S2, Figure S2).

The phylogenetic trees obtained from these sequences (Figure 2C and Figure S4, respectively) are also clearly not congruent with the phylogenetic history of the respective strains (Figure 2A). In addition, these trees show common features with the tree based on HPI genes (Figure 2B). Strains that are not grouped together in the phylogenetic tree of *E. coli* genomic genes (Figure 2A) are grouped together in the trees of HPI as well as the upstream and downstream HPI regions (Figure 2B, Figure 2C, and Figure S4). Furthermore, two strains of phylogenetic group D (ECOR 49 and 50) and two strains of phylogenetic group B2 (ECOR 61 and 62) have a similar deletion of the downstream regions and are accordingly grouped together in the trees of both the upstream region and the HPI (Figure 2B and 2C).

In order to statistically compare the structure of the trees independently of the branch length, we developed tests based on the path length difference (*pld*) metric [19]. We first performed permutation tests [20]–[22] between each pairwise distance tree matrices to show that they are statistically different from the null hypothesis of full incongruence. It turned out that all the elements of the pairwise distance tree matrices are significantly different from 0, indicating that the null hypothesis of incongruence can be rejected (p<0.001).

Then, as the hypothesis of incongruence is rejected, we compared the *pld* values between the strain chromosomal MLST reference tree and the HPI MLST, UR, and DR gene trees on one hand and the 6 individual housekeeping gene trees on the other. It shows that these *pld* are significantly higher in the first group than in the second one (Student t, t = 4.96,ddl = 7, p<0.002; Mann Whitney, p<0.02), leading to the conclusion that the trees of the 6 individual genes are statistically closer to the strain MLST than the HPI, UR or DR trees are.

**Figure 4:**
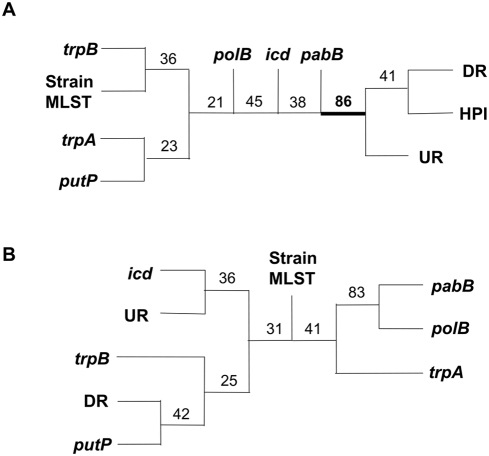
Tree representations of the distance matrix between gene tree structures. The trees represent the path length difference distance (*pld*) for (A) 30 *E. coli* HPI–positive strains, for which a DR region is complete and (B) 13 *E. coli* HPI–negative strains. Numbers are the bootstrap percentages (see text). In (A), the node, with its bootstrap value, delineating on one hand the six housekeeping genes and the strain MLST trees and on the other hand the HPI, UR, and DR trees are in bold.

To go further and compare statistically the degree of congruence between two tree distances, we applied the resampling method based on bootstrap described in the Material and Methods section. The tree in Figure 4A, performed with this approach, built from the data of the 30 *E. coli* HPI positive strains that have a complete UR and DR regions, clearly shows that the group of HPI, UR, and DR trees is supported (86%), and thus is statistically different from the strain MLST tree and from the individual housekeeping gene trees that show very low bootstrapping percentages (<45%).

These results indicate that (i) the 6 individual housekeeping gene trees and the strain MLST gene tree are closely related, as are the HPI UR and DR trees on their side; (ii) HPI, UR and DR tree structures are largely different from the 6 individual gene trees and from the strain MLST gene tree. We thus confirmed by this statistical resampling method the scenario inferred from the visual inspection of the trees from Figure 2 and Figure S4. Furthermore, the Ks of the UR and DR regions of the HPI positive strains are identical to the HPI gene ones, but different from the housekeeping genes (Figure 3) (Mann-Whitney, p = 0.0008).

To confirm the accuracy of the proposed scenario, we compared in 13 HPI negative strains representative of the genetic diversity of the species the phylogeny of the strains (based on the 6 housekeeping chromosomal genes) and the phylogeny of the UR and DR regions, as a control. The significant difference observed for the HPI positive strains between the *pld* values (see above) is not obtained for the 13 HPI negative strains (Student t, t = 1.05,ddl = 6, p<0.33; Mann Whitney, p<0.32). Conversely of what observed in the Figure 4A, Figure 4B shows that all the bootstrap values do not allow distinguishing UR and DR trees from the other gene trees.

To further detail the difference between, on one hand, the HPI region and its neighbouring UR and DR regions and, on the other hand, the rest of the chromosome, we performed several analyses with Clonal Frame [32]. This software allows a phylogenetic approach in the presence of recombination on MLST data and allows quantifying the relative importance of recombination and mutation. We first analyzed the chromosomal MLST sequences (6 housekeeping genes) of the 30 HPI-positive strains having both UR and DR regions, and found as estimated before [33] that the ratio of recombination to mutation is close to one: mean 1.8 [95% confidence interval (CI): 1 to 3.3]. We then performed the same analysis on the HPI MLST sequences and found no trace of recombination, the mean ratio of recombination to mutation being around 0.001 (95% CI: 0.00006 to 0.006), which reveals a recent acquisition of the HPI in the species such that no clear sign of recombination has yet occurred in that region of the genome. When the same approach was applied to the UR and DR regions, a small amount of recombination was found (mean ratio 0.57), but it is worth noting that the number of mutations present in these two genes is restricted, so estimates have to be taken with care as illustrated by the large confidence intervals (95% CI: 0.017 to 3.28) and the large fluctuations of likelihood observed (95% CI: −964 to −584). We then studied how the HPI with the UR and DR regions performed. Results were similar to that of HPI alone: almost no recombination was detected (mean ratio recombination to mutation: 0.0039, 95% CI: 0.00006 to 0.026), which suggest that both HPI and UR and DR regions have similar phylogenetic signals. Conversely, when we studied any combination including the chromosomal MLST and either UR and DR regions or HPI, or both groups, the program failed to converge. This suggests that the history of all those groups of loci are not compatible with the history of loci emerging from a simple population evolving under a constant rate of recombination as assumed in the Clonal Frame built-in model. Hence, the HPI is not compatible with the chromosomal MLST reflecting the history of the chromosome, and more interestingly, the regions neighboring the HPI locus are compatible with this region rather than with the rest of the chromosome even if they belong to the core genome of the species.

### Experimental Evidence That F Plasmids Can Transfer the HPI between *E. coli* Strains

Next, we sought to investigate *in vitro* the impact of large-scale DNA transfer and recombination events on the spread of the HPI among *E. coli* strains. Beside general transduction, which has a certain limitation in the size of the DNA-fragments to be transferred, the F-plasmid mediated DNA-transfer is likely to play a role in a “passive” horizontal transfer of genomic islands.

For this, the HPI-positive ExPEC strain NU14 was used as test strain in mating experiments [24]. In order to follow the transfer of the HPI, we first tagged the HPI of NU14 by insertion of a chloramphenicol resistance gene cassette using the method of Datsenko and Wanner [25]. This led to strain NU14-Cm, for which the exact location of the Cm-cassette at the right border of the HPI downstream of the *fyuA* gene was proven by PCR. By mating NU14-Cm with the laboratory *E. coli* strain XL-1 Blue MRF' (Stratagene, La Jolla, California) containing a tetracycline resistance mediating F-plasmid, the F' genotype was conferred on NU14-Cm. The resulting strain (NU14-CmF') served as donor in subsequent mating with *E. coli* K-12 strains AB 1157 and the respective *recA* mutant, AB1157 *recA^−^* as recipient (see Material and Methods). The HPI could be transferred from *E. coli* NU14-CmF' to *E. coli* AB1157 wild type strain at a frequency of 1×10^−9^. Interestingly, almost half of the transconjugants were tetracycline resistance indicating the presence of parts of the F-plasmid in these strains. By means of PCR amplifications using primers from different parts of the HPI we could demonstrate that the entire HPI was transferred to the respective recipient strain. No conjugational HPI transfer, however, could be observed using the *E. coli* AB1157 *recA* mutant strain.

**Figure 5:**
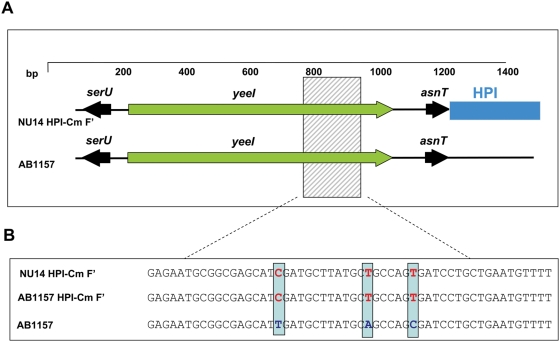
Conjugative F-Plasmid mediated transfer of the HPI. (A) Physical map and (B) partial sequence of the region upstream the HPI (UR) as found in donor (NU14 HPI-Cm F'), the transconjugant (AB1157 HPI-Cm F'), and the recipient (AB1157) strains. The partial sequence of *yeeI* gene given in the box reveals identical sequences in the donor and transconjugant strains, but not in the recipient strain indicating a transfer of the HPI together with the flanking upstream DNA region.

To determine whether the transferred HPI was integrated into the common HPI insertion site of the *E. coli* chromosome (*asnT* tRNA gene) further PCR amplifications of the *asnT* locus and the border of the HPI were performed. All of the tetracycline resistant transconjugants revealed an intact and unoccupied copy of the *asnT* tRNA locus indicating that the transferred HPI is either inserted in the recipient *E. coli* chromosome at a different site or is maintained episomally. In contrast, the tetracycline sensitive transconjugants carried the HPI at the *asnT* tRNA locus and no additional unoccupied copy of this locus was present in these strains. To determine whether the F-plasmid mediated DNA transfer affected the HPI together with the flanking DNA regions, the *yeeI* gene of the chromosomal DNA region immediate upstream of the HPI were PCR amplified and sequenced in the HPI-positive donor strain NU14 HPI-Cm F', the HPI-negative recipient strains (AB1157, AB1157 *recA*) and in all HPI-positive transconjugants. As shown in Figure 5, the sequence of the *yeeI* gene of *E. coli* NU14 HPI-Cm F' and AB1157 differed in distinct nucleotide positions enabling an assignment to the origin of the respective gene. In the tetracycline resistant HPI-positive *E. coli* AB1157 transconjugants two distinct copies of the *yeeI* gene could be detected representing the copy of both donor strain NU14 HPI-Cm F' and the recipient strain AB1157. In contrast, the tetracycline sensitive HPI-positive transconjugants of *E. coli* AB1157 revealed the presence a single copy of the *yeeI* gene, which was of the NU14 type. Thus, the original *yeeI* of AB1157 has been replaced by the transferred DNA region, suggesting a recombination event enclosing the flanking regions of the HPI and replacing the original DNA of the *asnT* tRNA locus as well as the *yeeI* gene. This indicates that the F-plasmid mediated HPI transfer indeed included the chromosomal DNA region flanking HPI (upstream HPI). Taken together, the transfer of the HPI within *E. coli* can be mediated by F plasmids and is dependent on the *recA* status of the recipient *E. coli* strain. In *recA*-proficient strains, the HPI and adjacent DNA regions are going to be integrated into the chromosome by recombination replacing the original DNA locus of the insertion site.

## Discussion

### Pathogenicity Islands and Horizontal Gene Transfer Influencing the Evolution of *E. coli*

One of the hallmarks of the bacterial evolution is the ability to acquire foreign DNA by HGT. Within the last decades HGT could be found to be a common phenomenon of bacteria, occurring even between very distantly-related ones [34]. It is thought to be a significant cause of increased drug resistance as well as the driving force for the evolution of bacterial virulence. Several different vehicles and mechanisms have been described for HGT crossing the species border. Beside phage mediated transduction and natural transformation of DNA, the plasmid mediated conjugative transfer is a powerful means for large scale DNA transfer. Chromosomal structures such as PAIs have been shown to extensively contribute to the evolution of bacterial pathogens by providing dynamic changes of the bacterial genome composition leading to a bacterial evolution in quantum leaps [35],[36]. Different mechanisms have been proposed for transfer of PAIs across the species border including phage transfer, mobilization by conjugative transposons and plasmids.

Phage transfer has been implicated in the transfer of some PAIs and PAI-like structures such as the staphyloccocal PAIs (SaPIs) encoding a type three secretion system (TTSS) [4]. It became clear from studies by Novick & coworkers that the SaPI is part of a defective bacteriophage of 15 kb, which can be excised and circulated by helper phages such as φ13 and φ80α [37]. After excision, the islands are transduced to other staphylococcal strains with high frequencies. This demonstrates the SaPI to be part of a group of mobile PAIs, which obviously derived from bacteriophages [4]. Another example for phage-mediated transfer of a PAI is the *Vibrio* pathogenicity island (VPI) of *V. cholerae*, which recently was shown to be mobilizable from one strain of *V. cholerae* to another by transduction. This island encodes several proteins with predicted sequences similar to those of proteins of bacteriophages or eukaryotic viruses as well as a protein that is highly homologous to the *E. coli* cryptic prophage (CP4-57) integrase [4],[38].

But also conjugative plasmids can mediate gene transfer between bacterial taxa in diverse environments. The ability to donate the F-type conjugative plasmid R1 greatly varies among enteric bacteria due to the interaction of the system that represses sex-pili formations of plasmids already harboured by a bacterial strain with those of the R1 plasmid [39]. The presence of efficient donors in heterogeneous bacterial populations can accelerate plasmid transfer and can spread by several orders of magnitude. However, the impact of plasmid mediated conjugative transfer for the transfer of pathogenicity islands between or within bacterial species has not been determined yet.

Although there are different examples for horizontal transfer of PAIs, relatively little is known about the mechanism underlying the spread of PAIs within a certain bacterial species. In this study, we therefore sought (i) to decipher the mechanisms underlying the distribution of a pathogenicity island within the *E. coli* species, and (ii) to analyze the impact of homologous recombination on the dissemination of this PAI. To address this issue we investigated the HPI as a model for transfer and evolution of an *E. coli* PAI. This HPI, first described in highly virulent *Yersinia* species [6],[40],[41], is a prototype of a PAI and encodes for the siderophore yersiniabactin mediated iron-uptake system. It is a well defined genomic island, which is widely distributed among different species and genera of the family *Enterobacteriaceae* [8],[42]. Interestingly, the HPI is not evenly distributed among the different *E. coli* phylogenetic groups. The results of the prevalence study of the HPI in ECOR strains confirmed previous findings that the HPI is present in all ECOR strains of B2 and D group [27], while HPI-positive strains are only sporadically found in phylogenetic groups A and B1. A comparable distribution pattern has been described for other *E. coli* PAIs [28]. This poses the intriguing question of how the HPI has been distributed among the phylogenetic groups within *E. coli* and what kind of mechanism regarding the genomic integration is associated with this distribution.

To answer these questions we first investigated structural features of the HPI in ECOR strains. As with the HPI in yersiniae, the HPI in *E. coli* is inserted in the *asnT* tRNA locus. Interestingly, in all except one ECOR strain the HPI is inserted at the *asnT* gene of the four *asn* tRNA gene copies found in *E. coli*. Previous reports on the HPI in yersiniae have demonstrated that all three copies of the *asn* genes are targeted as insertion site of the HPI [43]. In a single ECOR strain (ECOR31) the HPI is inserted at the *asnV* gene. This ECOR31-HPI displays a distinct large copy of the HPI encoding a functional mating pair formation and DNA processing system resembling conjugative plasmids [30]. For this large HPI an “active” transfer across the species border has been shown, which is facilitated by a combination of both phage related mobilization and conjugative properties of the HPI. Interestingly, after experimental transfer of this large mobilizable HPI, the HPI integrates at each of the tRNA copies of the recipient. Moreover, except the ECOR31-HPI all other HPIs of the ECOR strains revealed the presence of a unique deletion of the 3′-border downstream the *fyuA* gene affecting both the HPI and the neighbouring *E. coli* genome backbone (Figure S2). This is of particular interest, as the deletion results in loss of the direct repeat sequences flanking the HPI, hence rendering impossible the mobilization of the HPI by site specific recombination of the direct repeat sequences in a lambdoid phage type fashion. Thus, the HPI is fixed in the *E. coli* genome and spread of the HPI may therefore solely be due to (i) a vertical distribution (clonal), (ii) multiple independent insertion of the HPI into *E. coli* from another species or (iii) a transfer in a “passive” way by horizontal transfer of large DNA fragment carrying the HPI followed by homologous recombination and integration in the genome of the recipient.

The first hypothesis comprises a vertical clonal distribution of the HPI together with complete loss of HPI in certain branches of the ECOR strain phylogenetic tree. This is rather unlikely as the HPI-negative ECOR strains carry an intact *asnT* tRNA locus without any traces of the HPI or any deletions of the genomic backbone DNA neighbouring the HPI. The strongest argument against this hypothesis is the finding that the MLST trees derived from the HPI genes and the chromosomal backbone genes of the respective ECOR strain do not match (Figure 2 and Figure 4).

The second hypothesis of independent insertions of the HPI is true for the HPI of ECOR31 strain on one hand and all the other ECOR HPIs on the other. However, these other ECOR HPIs are inserted at the same tRNA copy, which is unlikely for multiple independent insertions. Moreover, all these HPIs reveal the same distinct deletion of the *fyuA* border, which includes parts of the neighbouring genome sequences (Figure S2). It appears extremely unlikely that different independent insertion of the HPI into *E. coli* result in such a distinct deletion pattern, which is not found after experimental transfer and integration of HPI of the ECOR31-type. A further observation that contradicts this hypothesis is the finding, that phylogenetic trees derived from the *E. coli* genomic DNA located directly upstream and downstream the HPI match with those of the HPI and not with the genomic backbone (housekeeping genes) of the respective ECOR strain (Figure 2 and Figure 4).

Thus, the results of this study clearly speak in favour of the third hypothesis that is a transfer in a “passive” way by horizontal transfer of a large DNA fragment carrying the HPI followed by homologous recombination and integration into the genome of the recipient.

Previously described self-transmissible conjugative elements can mobilize co-residing DNA either in *cis* or in *trans*. For example, conjugative plasmids like RP4 [44] can mediate transfer of mobilizable plasmids. These mobilizable plasmids typically encode an origin of transfer (*oriT*) and their own relaxase and nicking accessory proteins for interaction with *oriT*, but require a conjugative element to provide the mating pair formation functions for transfer [45]. Another transfer scenario is that a chromosome can acquire an *oriT* by integration of a conjugative element and thereby become mobilizable. For example, integration of the F plasmid in *E. coli* results in formation of the so-called Hfr (high frequency of recombination) strains, which can transfer large parts of their chromosomes at high frequency. In order to further support the third hypothesis of HPI distribution among ECOR strains, we carried out HPI-transfer experiments *in vitro* using a F plasmid as supporting vehicle. By this means we could demonstrate a conjugative transfer of the HPI mediated by the F-plasmid and a subsequent integration of the HPI into the chromosomal DNA of the recipient. This integration was strictly dependent on the presence of *recA* gene and included the DNA regions neighbouring the HPI, which were introduced from the donor into the recipient strain replacing the respective DNA of the recipient. This is in perfect agreement with the results obtained by the phylogenetic analyses of the ECOR strains.

One unexpected finding of this study was the extremely low level of synonymous and non-synonymous mutations in HPI genes and in the flanking regions of the different ECOR strains (Figure 3) as well as the absence of recombination within these regions. This hints towards a very recent integration of the HPI in the *E. coli* species as compared to the emergence of the *E. coli* species [46].

### Final Conclusion of HPI Distribution within *E. coli* and Theory of PAI Transfer in General

Taken together, the transfer and spread of PAIs in general and the HPI in particular may have occurred in two different steps: Firstly, the introduction of the HPI from a distantly related organism crossing the genus border. This may have been facilitated by phage-type, plasmid or ICE-type transfer with site-specific recombinations of attachment sites (tRNA genes). The HPI of the ECOR31 strain may represent an example of such an ancient HPI type. Secondly, the spread within the *E. coli* species across different phylogenetic groups after a transposition event, which may have involved a “passive” transfer by conjugative plasmids followed by homologous recombination with flanking DNA regions of the *E. coli* genomic backbone. The HPI of ECOR72 may represent an intermediate form, which is along with the ECOR31-HPI phylogenetically clearly distinct from the vast majority of *E. coli* HPIs (common type of *E. coli* HPI). The ECOR72-HPI, however, is inserted at the *asnT* gene and carries the deletion of the *fyuA* border, both of which are hallmarks of the common type of *E. coli* HPI. For the HPI it is of particular interest, that almost all *E. coli* HPIs appear to result from a single ancestor, which entered the *E. coli* species rather recently. The spread of the HPI must have occurred in a dramatically fast fashion, which may indicate a strong selective pressure. This led to the current situation of an extremely high (>80%) distribution of the HPI among all extraintestinal pathogenic *E. coli*.

## Supporting Information

Figure S1 Physical map of the High-Pathogenicity Island (HPI) depicting the location of the HPI-MLST PCR-primers as well as the respective PCR-fragments.
(0.01 MB PDF)

Figure S2 Physical map of the HPI-insertion site (*asnT* tRNA-locus) in the *E. coli* chromosome of HPI-negative (upper part) and HPI-positive ECOR strains (lower part). The location of the PCR-primers and the respective PCR-fragments of the region upstream the HPI (UR) as well as the region downstream the HPI (DR) are given. Note, that the region downstream the HPI is partially deleted in HPI-positive *E. coli* strains as indicated by spotted lines.
(0.02 MB PDF)

Figure S3 Genetic structure of the three different *E. coli* HPI-types: The first one found in the majority of HPI-positive *E. coli* strains (“normal” type), the second one of *E. coli* ECOR72 strain and the third of ECOR31 strain (“ECOR31-type”). The core region of the HPI encoding for the yersiniabactin siderophore system is highly conserved, whereas the *fyuA* border reveals distinct structural differences with a large deletion in HPIs of the “normal” type and ECOR72. The HPIs of these latter types are inserted at the *asnT* tRNA gene. However, the HPI of ECOR72 carries an insertion element (ISSfl14) at the *intB* border of the HPI leading to an interruption of the *intB* gene. The HPI of the ECOR31-type is rather distinct as it is inserted at the *asnV* copy of tRNA genes, reveals no deletion at the *fyuA* border, but instead an additional 35 kb DNA-region carrying the functional conjugative transfer system.
(0.01 MB PDF)

Figure S4 Phylogenetic unrooted tree using maximum likelihood procedure reconstructed from the region downstream the HPI (DR). The largest group of B2 strains is shown apart and the star indicates the midpoint rooting. The tree is built from the 30 of the previous 37 strains (Fig. 2) as (i) the downstream region was not studied in the EC31 UG and EC72 B1 strains (grey boxes in tree of Fig. 2A) because it is known to be distinct in these strains and (ii) the downstream region is partly deleted in 5 strains (EC48 D, EC49 D, EC50 D, EC61 B2 and EC62 B2). Bootstrap values calculated on 1000 replicated trees are shown if higher than 70%. Those strains showing identical grouping in this tree, the HPI MLST tree and the UR tree (Fig. 2B and C), but do not grouped together in the strain phylogeny tree (Fig. 2A) are boxed. Note that the strains EC49 D, EC50 D, EC61 B2 and EC62 B2 group (boxed) of the tree in Fig. 2C have a similar deletion of the downstream region. The ECOR strains are indicated by EC following by their number and the phylogenetic group to which they belong (A, B1, D, B2 and UG for ungrouped) [47].
(0.01 MB PDF)

Table S1 Oligonucleotide primers used in this study
(0.01 MB PDF)

Table S2 Main characteristics of the *E. coli* genes studied
(0.01 MB PDF)

Table S3 Likelihood estimation of the parameters carried out with PHYML (Guidon and Gascuel, 2003)
(0.01 MB PDF)
