# Supplementary figures and images for: Role of Intraspecies Recombination in the Spread of Pathogenicity Islands within the Escherichia coli Species
Source: PLoS Pathog. 2009 Jan 9;5(1):e1000257. doi: 10.1371/journal.ppat.1000257 (PMC2606025; doi:10.1371/journal.ppat.1000257)

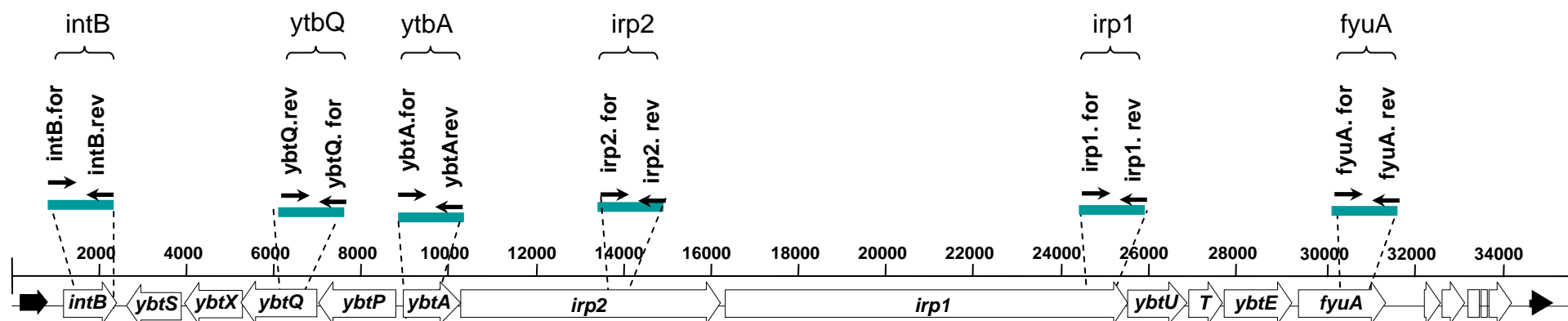

HPI-MLST sequences

Supplement: Figure S1 — Physical map of the High-Pathogenicity Island (HPI) depicting the location of the HPI-MLST PCR-primers as well as the respective PCR-fragments. (0.01 MB PDF) [file ppat.1000257.s001.pdf]

# ECOR

HPI negative

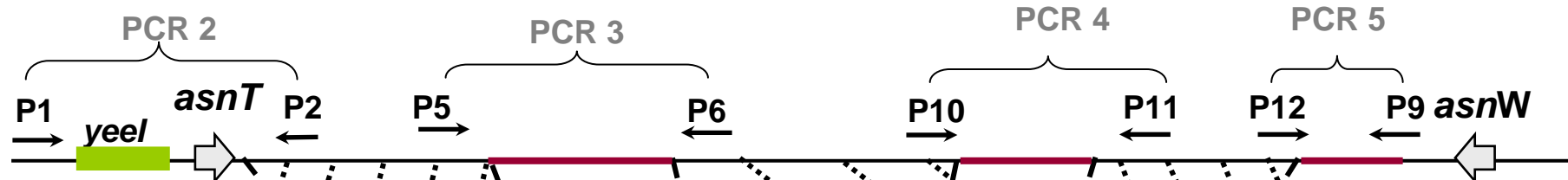

# ECOR

HPI positive

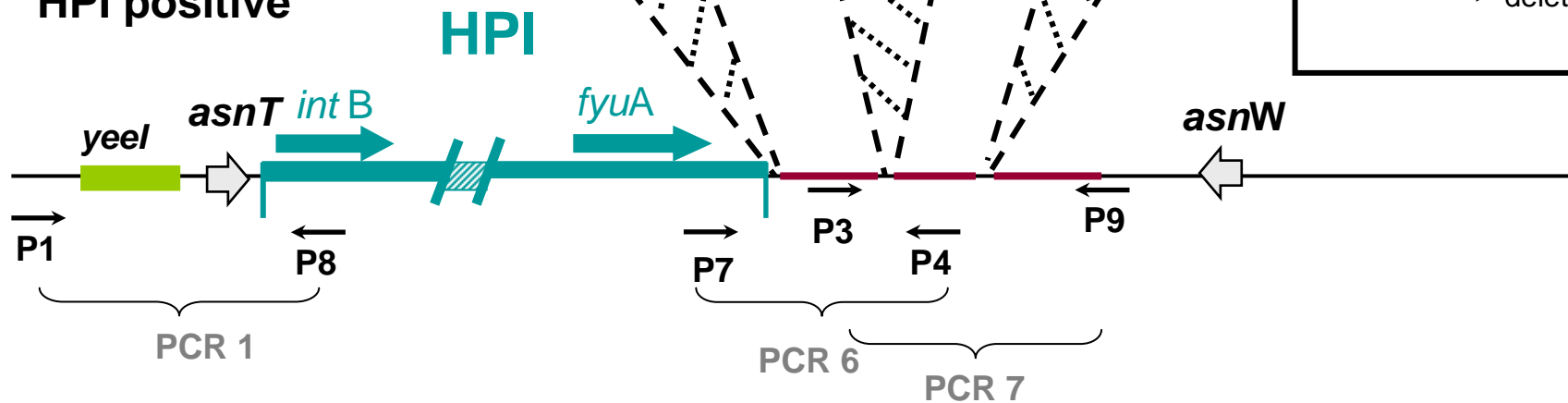

Supplement: Figure S2 — Physical map of the HPI-insertion site (asnT tRNA-locus) in the E. coli chromosome of HPI-negative (upper part) and HPI-positive ECOR strains (lower part). The location of the PCR-primers and the respective PCR-fragments of the region upstream the HPI (UR) as well as the region downstream the HPI (DR) are given. Note, that the region downstream the HPI is partially deleted in HPI-positive E. coli strains as indicated by spotted lines. (0.02 MB PDF) [file ppat.1000257.s002.pdf]

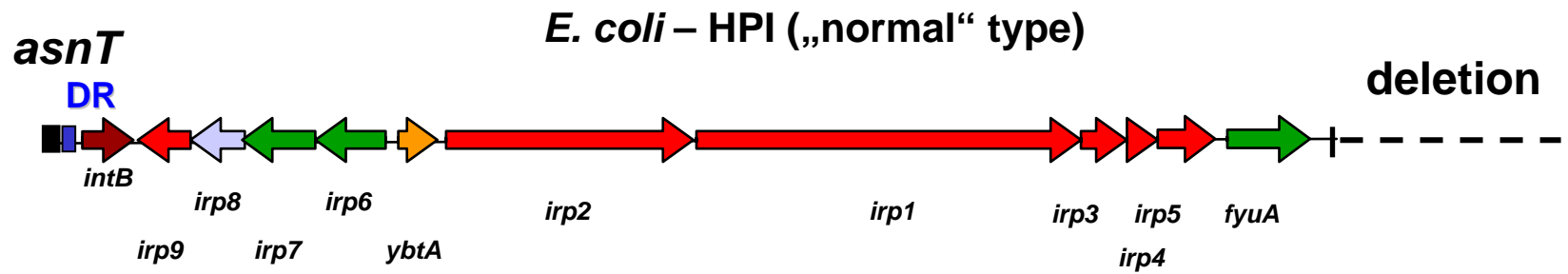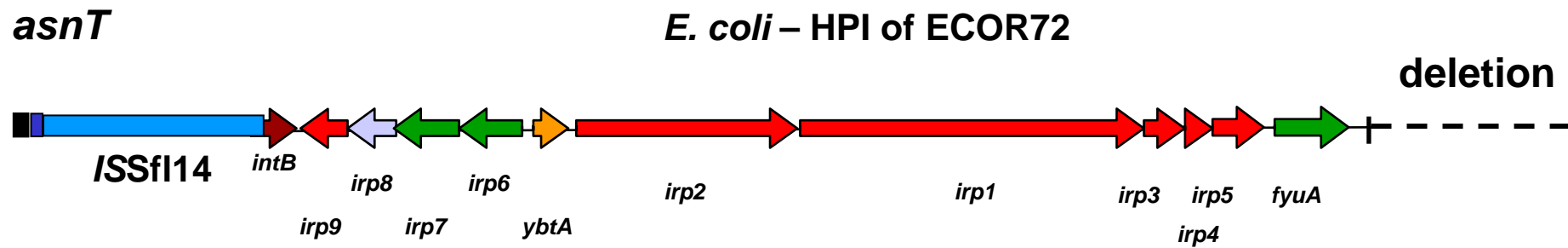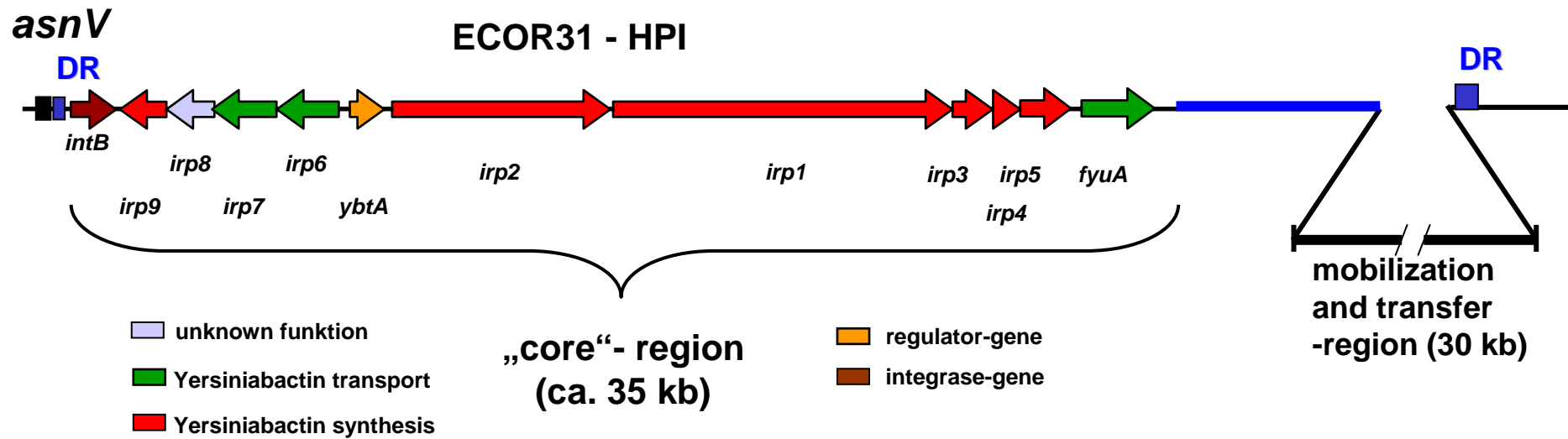

Supplement: Figure S3 — Genetic structure of the three different E. coli HPI-types: The first one found in the majority of HPI-positive E. coli strains (“normal” type), the second one of E. coli ECOR72 strain and the third of ECOR31 strain (“ECOR31-type”). The core region of the HPI encoding for the yersiniabactin siderophore system is highly conserved, whereas the fyuA border reveals distinct structural differences with a large deletion in HPIs of the “normal” type and ECOR72. The HPIs of these latter types are inserted at the asnT tRNA gene. However, the HPI of ECOR72 carries an insertion element (ISSfl14) at the intB border of the HPI leading to an interruption of the intB gene. The HPI of the ECOR31-type is rather distinct as it is inserted at the asnV copy of tRNA genes, reveals no deletion at the fyuA border, but instead an additional 35 kb DNA-region carrying the functional conjugative transfer system. (0.01 MB PDF) [file ppat.1000257.s003.pdf]

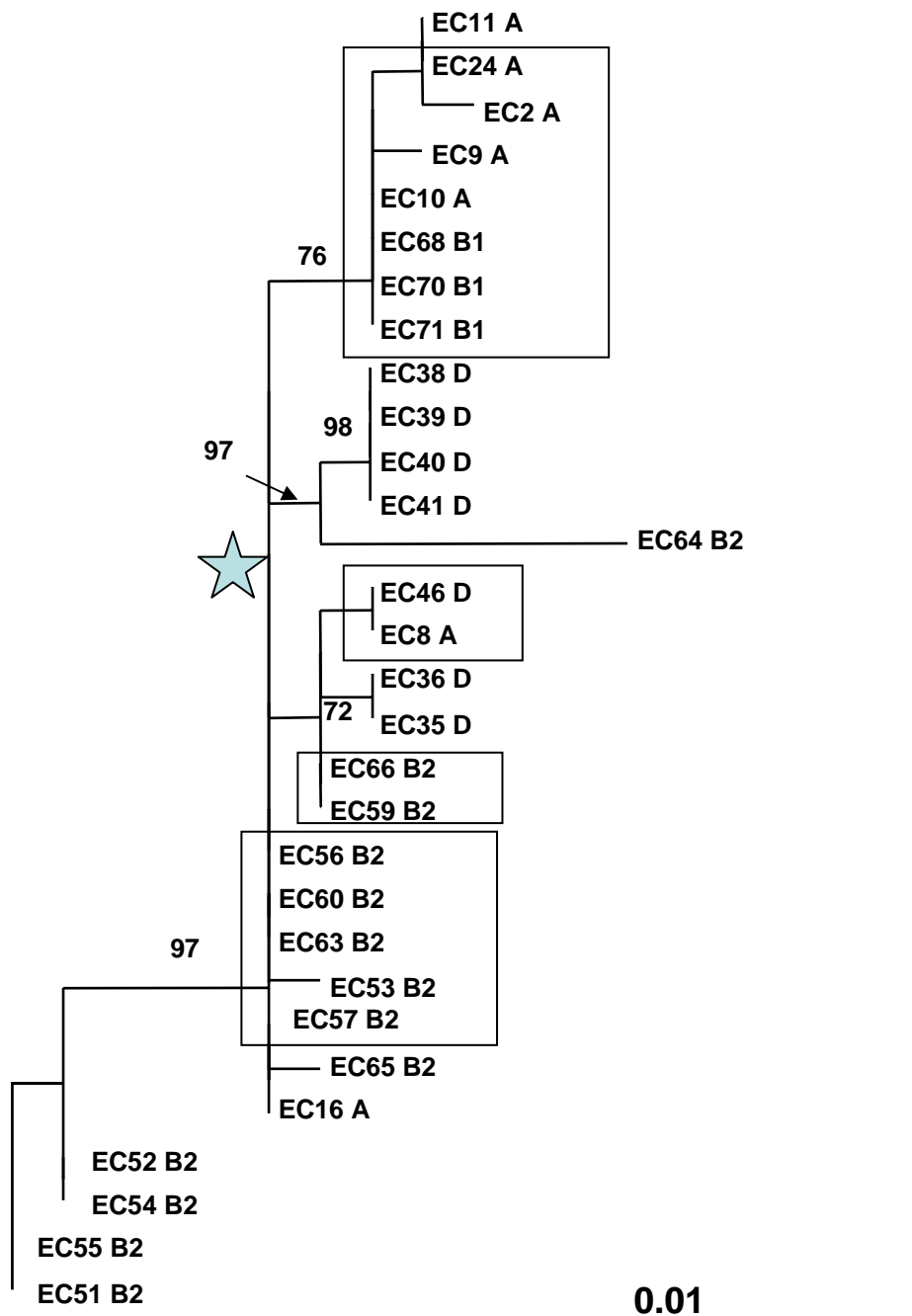

Supplement: Figure S4 — Phylogenetic unrooted tree using maximum likelihood procedure reconstructed from the region downstream the HPI (DR). The largest group of B2 strains is shown apart and the star indicates the midpoint rooting. The tree is built from the 30 of the previous 37 strains (Fig. 2) as (i) the downstream region was not studied in the EC31 UG and EC72 B1 strains (grey boxes in tree of Fig. 2A) because it is known to be distinct in these strains and (ii) the downstream region is partly deleted in 5 strains (EC48 D, EC49 D, EC50 D, EC61 B2 and EC62 B2). Bootstrap values calculated on 1000 replicated trees are shown if higher than 70%. Those strains showing identical grouping in this tree, the HPI MLST tree and the UR tree (Fig. 2B and C), but do not grouped together in the strain phylogeny tree (Fig. 2A) are boxed. Note that the strains EC49 D, EC50 D, EC61 B2 and EC62 B2 group (boxed) of the tree in Fig. 2C have a similar deletion of the downstream region. The ECOR strains are indicated by EC following by their number and the phylogenetic group to which they belong (A, B1, D, B2 and UG for ungrouped) [47]. (0.01 MB PDF) [file ppat.1000257.s004.pdf]
