# Supplementary material for: Role of Intraspecies Recombination in the Spread of Pathogenicity Islands within the Escherichia coli Species
Source: PLoS Pathog. 2009 Jan 9;5(1):e1000257. doi: 10.1371/journal.ppat.1000257 (PMC2606025; doi:10.1371/journal.ppat.1000257)
Supplement: Table S1 — Oligonucleotide primers used in this study (0.01 MB PDF) [file ppat.1000257.s005.pdf]

Table S1. Oligonucleotide primers used in this study

locus	PCR / gene	forward-primer	sequence	reverse-primer	sequence	fragment length
**HPI-MLST PCRs**						
	intB	intB.for	5’-CCAGTCAGAGGAGCCAATTTT-3’	intB.rev	5’-CCGGGAGAAATTAGCGAGCAT-3’	1,7 kb
	ybtQ	ybtQ.for	5’-GCGGCAGGGCGGGTGAAATGA-3’	ybtQ.rev	5’-TGCGACGGGAAGCGGTGCTAT-3’	650 bp
	ybtA	ybtA.for	5’-GCCATAGCTAAAATAACCTTCCT-3’	ybtA.rev	5’-CCGGTAACAGCGAATCCTGAGAT-3’	1,5 kb
	irp2	irp2.for	5’-CTCCGCAGAACAGGTAGCCGA-3’	irp2. rev	5’-GCCAGATAATGTCCGCCGAGT-3’	637 bp
	irp1	irp1.for	5’-CCATCTACAGGCGGCTACGTC-3’	irp1.rev	5’-GTCTCCCGGCGGCATACACCA-3’	654 bp
	fyuA	fyuA.for	5’-CTACGACATGCCGACAATGCC-3’	fyuA.rev	5’-TGCTTCCCGCGCCATAACGTG-3’	670 bp
**„upstream HPI“**						
in HPI-positive strains	**1**	P1	5’-GGAAGTCGGGAGATAAGTCATTGATAAAGTGGC-3’	P8	5’-CCAATTTTTCCCGATGCGTAG-3’	1,3 kb
in HPI-negative strains	**2**	P1	5’-GGAAGTCGGGAGATAAGTCATTGATAAAGTGGC-3’	P2	5’-GGCCGATTCCAACGCTCCAAG-3’	1,2 kb
**“downstream HPI”**						
in HPI-negative strains	**3**	P5	5’-GCGGCAGCACAAGGTGTGGTAAA-3’	P6	5’-CTAAGCCGTTGTTAATCTGCGTA-3’	600 bp
	**4**	P10	5’-ACCAACCACGACGGCAATTTTTA-3’	P11	5’-AACCGCCTTGTCCGAGAGTAAAG-3’	600 bp
	**5**	P12	5’-ACTTTGATTGCCGATGCTGGTAC-3’	P9	5'-CATGGTGACCTCGGCTACTCCCT-3’	650 bp
**“downstream HPI”**						
in HPI-positive strains	**6**	P7	5’-TGGCAACATCCTACTATCGGTC-3’	P4	5’-GACGTTCGGCACATAGGTCA-3’	
	**7**	P3	5’-CACGTGGATGGGCCTCTTCTCAG-3’	P9	5'-CATGGTGACCTCGGCTACTCCCT-3’	1 kb
