# Supplementary material for: Role of Intraspecies Recombination in the Spread of Pathogenicity Islands within the Escherichia coli Species
Source: PLoS Pathog. 2009 Jan 9;5(1):e1000257. doi: 10.1371/journal.ppat.1000257 (PMC2606025; doi:10.1371/journal.ppat.1000257)
Supplement: Table S2 — Main characteristics of the E. coli genes studied (0.01 MB PDF) [file ppat.1000257.s006.pdf]

**Table S2**. Main characteristics of the *E. coli* genes studied

| Genes | Number of nucleotides | HPI positive strains | | HPI negative strains | |
|---|---|---|---|---|---|
| | | Number of strains | Number of informative sites for parsimony | Number of strains | Number of informative sites for parsimony |
| *icd* | 1166 | 37 | 68 | 13 | 25 |
| *putP* | 892 | 37 | 88 | 13 | 52 |
| *polB* | 995 | 37 | 96 | 13 | 39 |
| *trpA* | 727 | 37 | 60 | 13 | 16 |
| *trpB* | 1138 | 37 | 83 | 13 | 35 |
| *pabB* | 1003 | 37 | 59 | 13 | 7 |
| Strain MLST | 5919 | 37 | 454 | 13 | 174 |
| *int* | 789 | 37 | 8 | | |
| *ybtQ* | 594 | 37 | 1 | | |
| *ybtA* | 957 | 37 | 7 | | |
| *irp2* | 585 | 37 | 6 | | |
| *irp1* | 594 | 37 | 9 | | |
| *fuyA* | 600 | 37 | 3 | | |
| HPI MLST | 4119 | 37 | 34 | | |
| UR | 837 | 37 | 19 | 13 | 3 |
| DR | 1162 | 30 | 12 | 13 | 63 |
