# Supplementary material for: Role of Intraspecies Recombination in the Spread of Pathogenicity Islands within the Escherichia coli Species
Source: PLoS Pathog. 2009 Jan 9;5(1):e1000257. doi: 10.1371/journal.ppat.1000257 (PMC2606025; doi:10.1371/journal.ppat.1000257)
Supplement: Table S3 — Likelihood estimation of the parameters carried out with PHYML (Guidon and Gascuel, 2003) (0.01 MB PDF) [file ppat.1000257.s007.pdf]

Table S3: Likelihood estimation of the parameters carried out with PHYML (Guidon and Gascuel, 2003). For each sequence, the most parsimonious model is selected following the Akaike criterium. Upper part: base frequencies. Lower part: substitution rates (tv: transversion; ti transition), G parameter of the Gamma distribution of the heterogeneity of substitution rates among sites (eight discrete classes), and proportion of invariant sites, p-inv.

	**A**	**C**	**G**	**T**
6 genes (1)	0,227	0,257	0,281	0,235
icd	0,250	0,250	0,250	0,250
pabB	0,250	0,250	0,250	0,250
polB	0,223	0,261	0,288	0,229
putP	0,179	0,230	0,328	0,263
trpA	0,213	0,269	0,275	0,243
trpB	0,234	0,268	0,287	0,211
UR	0,246	0,217	0,237	0,300
HPI	0,219	0,283	0,284	0,214
DR	0,295	0,246	0,253	0,206

		tv	ti	tv	tv	ti	tv		
		a	b	c	d	e	f		
	model (2)	**AC**	**AG**	**AT**	**CG**	**CT**	**TG**	**p-inv**	**G**
6 genes (1)	**GTR+I+G**	0,475	4,075	1,082	0,999	5,042	[1]	0,517	0,163
icd	**TIM1ef+I+G**	1,000	2,754	1,839	1,839	10,270	[1]	0,760	0,493
pabB	**TrNef+G**	1,000	5,111	1,000	1,000	3,242	[1]		0,062
polB	**GTR+G**	0,376	5,786	0,887	0,922	3,655	[1]		0,090
putP	**TrN+G**	1,000	2,962	1,000	1,000	5,791	[1]		0,108
trpA	**HKY+I**	kappa = 5.643 (ti/tv = 2.799)						0,856	
trpB	**GTR+G**	0,127	3,186	1,171	1,011	4,814	[1]		0,048
UR	**TrN+I**	1,000	47,178	1,000	1,000	11,365	[1]	0,926	
HPI	**TPM1uf+I+G**	1,000	4,954	0,605	0,605	4,954	[1]	0,323	0,010
DR	**HKY**	kappa = 5.6107 (ti/tv = 2.839)							

(1) icd+pabB+polB+putP+trpA+trpB concatenated
(2) following Posada's nomenclature (jModelTest: Phylogenetic Model Averaging. Mol. Biol. Evol., July 2008; 25: 1253 - 1256).
